# Diagnostic performance of a computer-assisted diagnostic system: sensitivity of BONENAVI for bone scintigraphy in patients with disseminated skeletal metastasis is not so high

**DOI:** 10.1007/s12149-020-01435-0

**Published:** 2020-01-22

**Authors:** Mitsuru Koizumi, Kazuki Motegi, Masamichi Koyama, Mitsutomi Ishiyama, Takashi Togawa, Tomoko Makino, Yukiko Arisaka, Takashi Terauchi

**Affiliations:** 1 Department of Nuclear Medicine, Cancer Institute Hospital, 3-8-31 Ariake, Koto-ku, Tokyo, 135-8555 Japan; 2 Graduate School of Health Science, Department of Medical Radiological Technology, Faculty of Health Science, Kyorin University, Tokyo, Japan

**Keywords:** Bone scintigraphy, BONENAVI, ANN, Disseminated skeletal metastasis, Prostate cancer, Gastric cancer, Breast cancer

## Abstract

**Purpose:**

Bone scintigraphy (BS) of disseminated skeletal metastasis is sometimes misinterpreted as normal. The use of computer-assisted diagnosis (CAD) may resolve this problem. We investigated the performance of a CAD system, BONENAVI, in the diagnosis of disseminated skeletal metastasis.

**Methods:**

Cases of disseminated skeletal metastasis were selected from a BS log. These patients’ BSs were analyzed by BONENAVI to obtain an artificial neural network (ANN) and bone scan index (BSI). Clinical features (type of primary cancer, CT type, and BS type) were compared with the BONENAVI (ANN and BSI) results. The BS findings (diffuse increased axial skeleton uptake, inhomogeneity of uptake, proximal extremity contrast, and degree of renal uptake) and ANN or BSI were evaluated. Then, negative ANN patients were presented.

**Results:**

Fifty-four patients were diagnosed as having disseminated skeletal metastasis. Regarding the primary cancers, 12 had prostate cancer, 16 gastric cancers, 16 breast cancers, and 10 miscellaneous cancers. Total sensitivity of ANN (≥ 0.5) was 76% (41/54). ANN values correlated with the BS type among clinical features. Diffuse increased axial skeleton uptake was mostly correlated with ANN of the BS findings.

**Conclusion:**

The BONENAVI CAD system was partially helpful in diagnosing disseminated skeletal metastasis, but the sensitivity of BONENAVI was not sufficient and underestimated the disseminated skeletal metastasis. Further improvement of this CAD system is necessary to improve the detectability of disseminated skeletal metastasis.

## Introduction

The diagnosis of disseminated skeletal metastasis by bone scintigraphy (BS) is usually easily established when typical findings are present. However, the BS pattern is easily misinterpreted as normal unless there is an absence of renal uptake, or the presence of diminished activity in the bones of the appendicular skeleton, and when a high ratio of bone to soft tissue activity is recognized [1, 2].

Until recently, BS was interpreted visually. However, considering the need for an appropriate quantitative approach for BS, computer-assisted diagnostic (CAD) software for BS was developed and evaluated [3–5]. A Japanese version of this software is BONENAVI [6]. We previously published a formal report on the development and clinical evaluation of a revised version, called BONENAVI II [7]. Although the sensitivity and specificity of BONENAVI are reportedly good for skeletal metastasis [8, 9], we also reported that skeletal metastasis from prostate cancer with a small tumor burden often shows false-negative results in CAD (BONENAVI II) analysis [10].

Our clinical questions are how the BONENAVI performed in patients with disseminated skeletal metastasis, and what factors, if any, caused the failures (false-negative results) in the diagnosis of the skeletal metastasis by BONENAVI in disseminated skeletal metastasis patients.

Therefore, we tried to clarify the sensitivity of BONENAVI II in patients with disseminated skeletal metastasis. We also tried to determine the clinical features or BS features that cause mismatches in the skeletal involvement and negative CAD analysis.

## Patients and methods

### Patients

We selected patients with disseminated skeletal metastasis from the BS log from January 2013 to August 2019 at the cancer institute hospital, Tokyo, Japan. From the log, 63 patients were listed. These patients were diagnosed on BS to have definite, suspected or not-ruled-out disseminated skeletal metastasis. We reviewed the BS and other clinical data, including computed tomography (CT), magnetic resonance imaging (MRI) and/or fluoro-deoxy glucose positron emission tomography with CT (FDG-PET/CT). Fifty-four patients were confirmed to have disseminated skeletal metastasis; that is, skeletal metastatic lesions had spread to nearly the entire axial skeleton, equivalent to the extent of the disease (EOD) = IV [11]. These 54 patients were selected for this study. Nine patients were excluded from this study because 8 had skeletal metastasis, but the spread of the disease did not meet the criteria of the term ‘disseminated’, while the other one patient did not show skeletal metastasis.

The diagnosis of disseminated skeletal metastasis was established clinically; that is, no skeletal metastatic lesion was diagnosed histologically. The diagnosis of disseminated skeletal metastasis was established and confirmed using at least two diagnostic modalities (from BS, CT, MRI, and FDG-PET/CT).

Primary cancer sites were variable: 12 patients had prostate cancer, 16 gastric cancers, 15 breast cancers, and 10 miscellaneous cancers (2 pulmonary cancers, 2 esophageal cancers, 1 maxillary cancer, 1 bladder cancer, 1 pancreatic cancer, 1 pancreas neuro–endocrine tumor, 1 bile duct cancer, and 1 synovial sarcoma).

This retrospective study was approved by the local institutional review board.

### Bone scintigraphy

BS was performed about 3 h (range 2–4 h) after injection of 740 MBq of ^99m^Tc-MDP. After obtaining anterior and posterior whole-body scans, local images, the pelvic axial view, were also acquired. Whole-body (anterior and posterior) images were taken using E-CAM (Canon), Infinia (GE) or Intevo (Siemens) using 15–20-cm /min scan speed with a 256 × 1024 matrix size. After taking whole-body images, static studies were conducted using 6–8 million counts/view with a 512 × 512 matrix size.

BS findings were reviewed, and the presence of the characteristics of disseminated skeletal metastasis based on each BS clinical report was recorded. BS findings of disseminated skeletal metastasis were characterized by intense uptake throughout the skeleton; faint or absent renal uptake; patchiness (inhomogeneity) of uptake, particularly in the ribs; and the presence of diminished activity in the appendicular skeleton [1, 2]. These findings were changed to: 1. diffuse axial skeleton radionuclide (RN) uptake (positive, weak positive, or negative), 2. inhomogeneity of RN uptake (inhomogenous, partially inhomogenous, or no), 3. decreased appendicular skeleton uptake or relatively increased uptake in a proximal extremity (yes or no), and 4. absent kidney sign (absent kidney uptake, weak renal imaging, or normal renal uptake).

Based on the above findings, BS visual interpretations were classified into (a) definite BS type: definite super bone scan; diffuse increased axial skeleton RN uptake irrespective of other findings, (b) suspected BS type: suspected disseminated bone metastasis (definite skeletal metastasis but not a typical super scan); weak or no diffuse axial skeleton RN uptake and inhomogeneity of RN uptake, irrespective of other findings, (c) not-ruled-out BS type: not-ruled-out disseminated skeletal metastasis; other than (a) or (b) above; weak or no diffuse axial skeleton uptake, partial or no inhomogeneity, and increased proximal extremity uptake or absent or weak renal uptake.

### BONENAVI

BONENAVI analysis was performed as previously reported [7]. Briefly, the CAD system, BONENAVI version 2.1.7 (FUJIFILM RI Pharma Co., Ltd., Tokyo, Japan), was used to analyze BS scans using anterior and posterior whole-body images. This BONENAVI system shows two imaging markers: an artificial neural network (ANN) and a bone scan index (BSI). The ANN value indicates the probability of having skeletal metastasis. It has an ANN range of 0–1, where “0” means no possibility of skeletal metastasis, and “1” means a high suspicion of having skeletal metastasis. The BSI indicates the tumor metastatic burden (proportion of bone metastatic area to whole-body skeleton). In the present study, both the ANN and BSI values were used.

### CT and classification

CT scans were obtained with a 2-mm thickness at 5-mm intervals.

The CT morphological classification of skeletal metastasis included the categories osteoblastic, osteolytic, mixed osteoblastic and osteolytic, and intertrabecular (invisible on CT). This classification was independently performed by two radiologist-nuclear physicians, and discordant cases were decided through discussion. The diagnostic confirmation of disseminated skeletal metastasis was made by the agreement of two physicians using at least two imaging modalities (BS, CT, MRI, FDG-PET/CT).

### Analysis process

1. We observed the relationships among the types of primary cancer, CT types, and BS types.
2. We investigated the relationship between BONENAVI: ANN ≥ 0.5 and the types of primary cancer, CT types or BS types.
3. We analyzed the relationship between BONENAVI (ANN and BSI values) and clinical features; the types of primary cancer, CT types or BS types by one-way analysis of variance (one-way ANOVA).
4. To clarify the BS factors that were related to BONENAVI (ANN and BSI values), the relationship of ANN values and BS findings (the presence of diffuse increased skeleton uptake, inhomogeneity, increased proximal extremity uptake, or absent kidney sign) was investigated by one-way ANOVA. Using BONENAVI: ANN ≥ 0.9 as a dependent factor, multivariate logistic analysis was also employed to determine which factor was related to a high ANN value. We initially used ANN ≥ 0.5; however, there was no patient whose diffuse axial skeleton uptake was below 0.5 (ANN), and analysis was not properly performed. Therefore, we selected ANN ≥ 0.9 as the dependent factor instead of ANN ≥ 0.5.
5. We also observed the relationship between BSI values and BS findings by one-way ANOVA.
6. We added the list of patients with various factors who showed negative ANN (ANN < 0.5) despite the presence of disseminated skeletal metastasis.

### Statistical methods

Statistical analysis software (SPSS version 24; IBM, Armonk, NY, USA) was used. A value of *p* < 0.05 was considered to indicate statistical significance. For one-way ANOVA, Levene’s test for homogeneity of variance was performed first. When the homogeneity of variance was assumed, a *t *test was followed by Tukey’ test. When the homogeneity was not assumed, Welch’s test was followed by the Games–Howell test. For contingency table analysis, if more than 20% of cell boxes was expected to number < 5, Fisher’s exact test was used. Pearson’s Chi-square test was applied to the others.

The multivariate logistic regression analysis was carried out using ANN ≥ 0.9 as a dependent factor both by forward and backward stepwise analysis using a likelihood ratio.

## Results

### Patient profiles

Totally 54 patients were diagnosed clinically to have disseminated skeletal metastasis. Primary cancer sites were variable: 12 patients had prostate cancer, 16 gastric cancers, 15 breast cancers, and 10 miscellaneous cancers (2 pulmonary cancers, 2 esophageal cancers, 1 maxillary cancer, 1 bladder cancer, 1 pancreatic cancer, 1 pancreas neuro-endocrine tumor, 1 bile duct cancer, and 1 synovial sarcoma).

Twenty-five CT patterns were osteoblastic type, 19 mixed type, and 10 intertrabecular type. Twenty-eight BS types were of definite type, 16 were of suspected type, and 10 were of not-ruled-out type. The relationships among these factors are summarized in Table 1.

**Table 1:** Patients’ Demographics

1-A	CT type			Total	*p* value**
	osteoblastic	Mixed	Intertrabecular		
Primary cancer					0.001
Prostate cancer	11*	1	0	12	
Gastric cancer	9	4	3	16	
Breast cancer	3	10	3	16	
Miscellaneous cancers	42	4	4	10	
Total	25	19	10	54	

1-B	BS type			Total	*p* value**
	Typical	Suspected	Not-ruled-out		
Primary cancer					0.01
Prostate cancer	10	1	1	12	
Gastric cancer	10	2	4	16	
Breast cancer	6	8	2	16	
Miscellaneous cancers	2	5	3	10	
Total	28	16	10	54	

1-C	BS type			Total	*p* value**
	Typical	Suspected	not-Ruled-out		
CT type					0.008
Osteoblastic	14	5	6	25	
Mixed	12	7	0	19	
Intertrabecular	2	4	4	10	
Total	28	16	10	54	

*Number of patients

**p value was calculated by Fisher's exact test

Disseminated skeletal metastasis from prostate cancer tended to have a high percentage of CT osteoblastic type (11/12) and BS definite type (10/12).

Disseminated skeletal metastasis from gastric cancer showed the next highest ratios as CT osteoblastic type (9/16) and BS definite type (10/16).

Breast cancer showed the lowest ratios among the three most frequent cancers as CT osteoblastic type (3/16) and BS definite type (6/16).

In all patients, CT osteoblastic (56%, 14/25) and mixed (63%, 12/19) types showed similar ratios to the definite BS type, and the CT intertrabecular pattern showed a low ratio to the definite BS type (20%, 2/10). There were no patients with osteolytic type who showed disseminated skeletal metastasis.

### Relation to BONENAVI ANN sensitivity

**Fig. 1:**
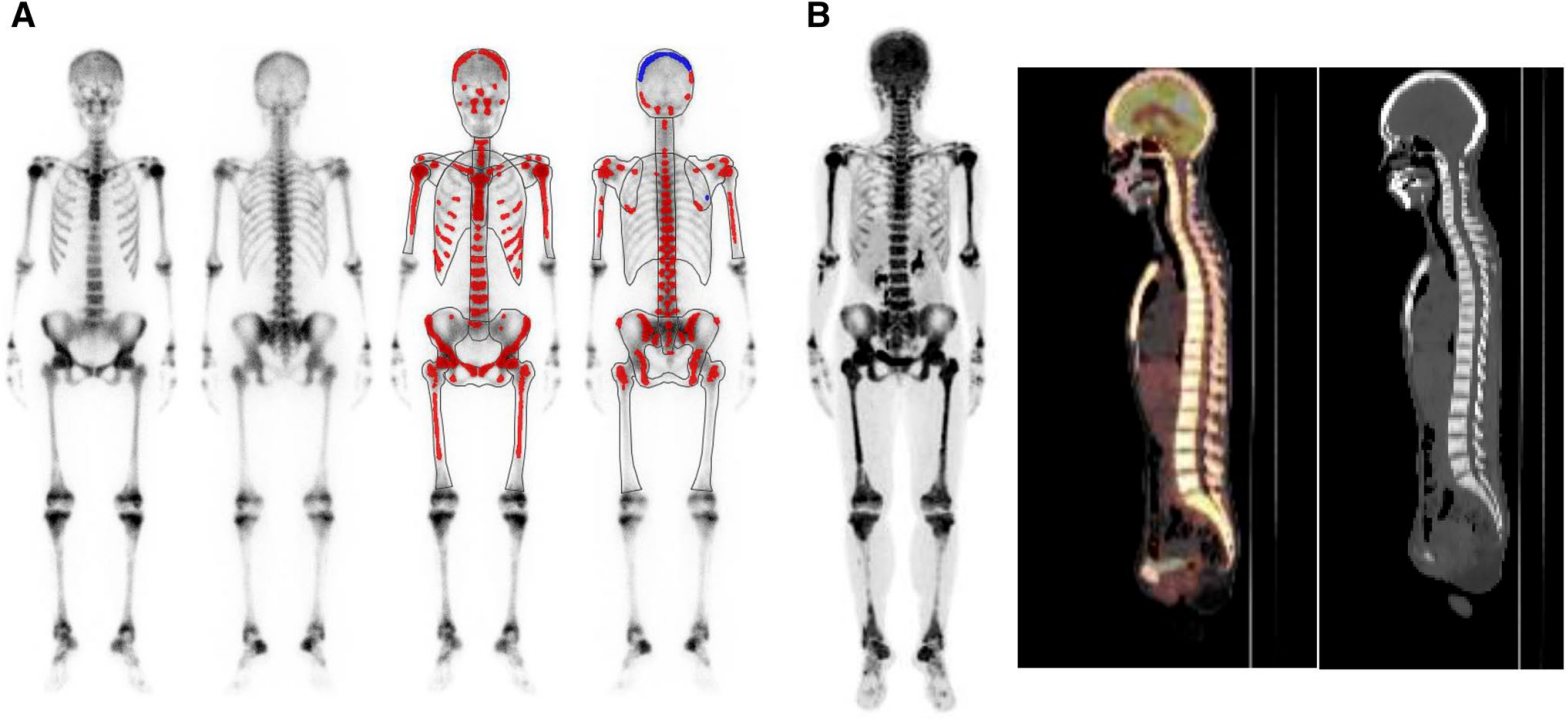
This 47-year-old female had a history of gastric cancer (poorly differentiated signet ring cell) and underwent surgery 9 years prior to this study. She was diagnosed as having skeletal metastasis 6 months prior to this study. Her alkaline phosphatase (ALP) and tumor marker (carcinoembryonic antigen: CEA) increased (AL* p* = 10,338 U/l and CEA = 18.3 ng/ml), and she underwent bone scintigraphy (BS) because of increasing back pain. Anterior and posterior views of whole-body imaging BS (left) and BONENAVI processed images with artificial neural network (right) (**a**). Diffuse skeleton increased radionuclide uptake was shown with absent kidney sign on BS (BS definite type). The interpretation of BS was “disseminated skeletal metastasis, so called super bone scan”. BONENAVI analysis showed ANN = 1.0 and BSI = 14.386. Fluoro-deoxy glucose positron emission tomography with computed tomography (FDG-PET/CT) images are shown **b** a maximum intensity projection (MIP) image (left), a fusion image of FDG-PET and CT (sagittal) (center), and a sagittal CT image (right). Systemic bone/bone marrow FDG uptake was noticed, and the osteoblastic nature of skeletal metastasis was evident

**Fig. 2:**
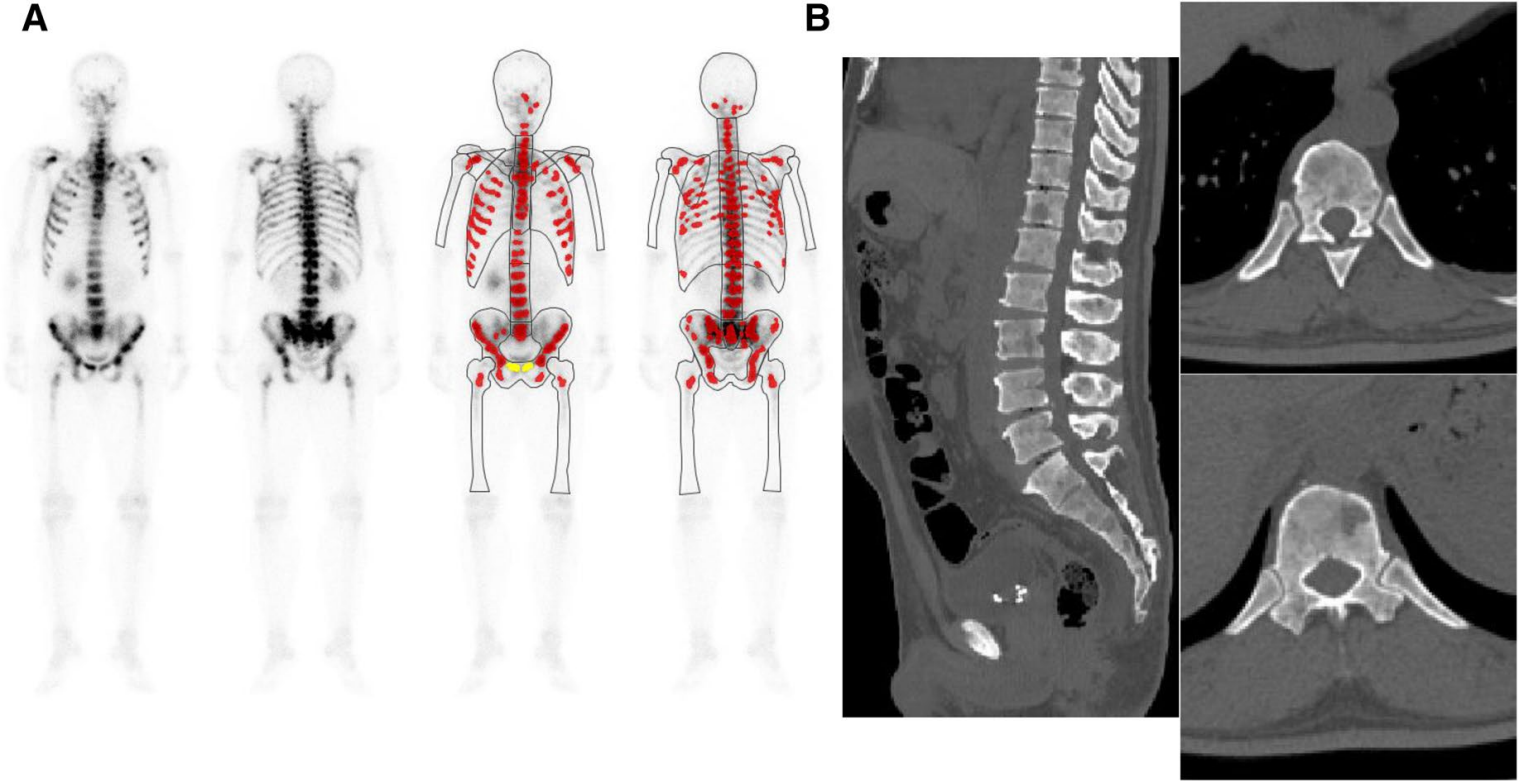
This 46-year-old male was diagnosed as having diffuse infiltrating type-4 advanced gastric cancer (poorly differentiated signet ring cell) 2 months prior to this study. Biochemical analysis showed an elevation of alkaline phosphatase (AL* p* = 4725 U/l); therefore, bone scintigraphy (BS) was performed. Anterior and posterior views of whole-body BS (left) and BONENAVI processed images with artificial neural network (right) **a** show diffuse increased radionuclide (RN) axial skeleton uptake; inhomogeneity of RN uptake, especially in ribs; increased proximal extremity uptake (or contrast of proximal femur and distal portion); and weak renal uptake. The interpretation of BS was “disseminated skeletal metastasis” (BS definite type). BONENAVI analysis showed ANN = 1.0 and BSI = 11.043. CT images of sagittal (left), and axial (right, upper and lower) slices **b**. Mainly osteoblastic and partially osteolytic lesions were disseminated in the whole skeleton

Figures 1 and 2 show patients with disseminated skeletal metastasis who showed definite BS types and positive BONENAVI results (high ANN and BSI).

**Fig. 3:**
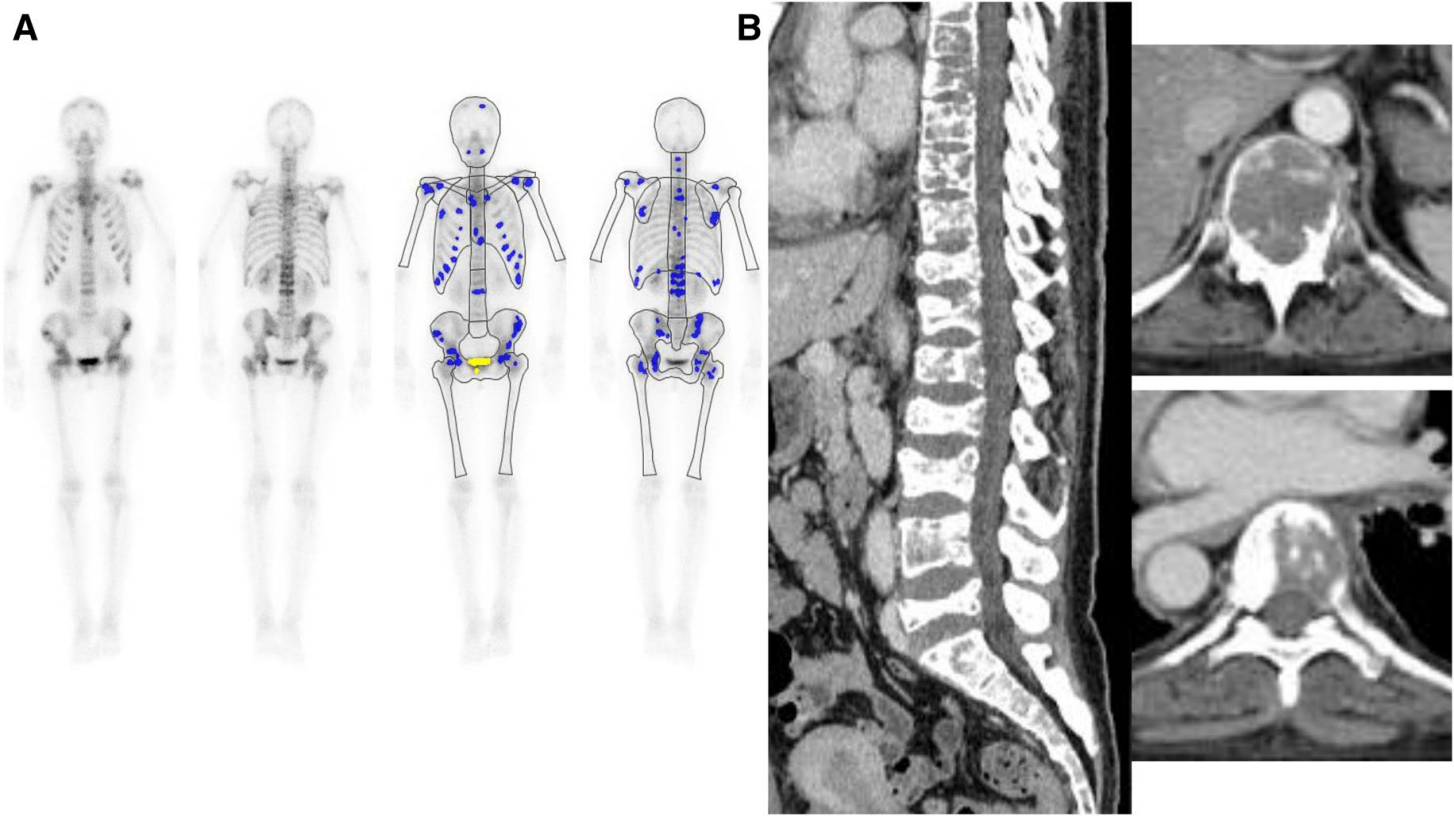
This 34-year-old female was diagnosed with advanced left breast cancer (invasive ductal carcinoma) with skeletal metastasis. Despite hormone therapy, she had progressive back and right chest pain. Her ALP and CEA values were high (AL* p* = 501 U/l and CEA = 99.0 ng/ml). Bone scintigraphy (BS) was performed. Anterior and posterior views of whole-body BS (left) and BONENAVI processed images with artificial neural network (right) **a** show weakly increased radionuclide (RN) axial skeleton uptake; inhomogeneity of RN uptake, especially in vertebrae and ribs; increased proximal extremity uptake (or contrast of proximal femur and distal portion); and normal renal uptake were shown. The interpretation of BS was “definite multiple skeletal metastasis but disseminated spread was not certain” (BS suspected type). However, BONENAVI analysis showed ANN = 0.0 and BSI = 0.0. This is a false-negative BONENAVI case. CT images of sagittal (left), and axial (right, upper and lower) slices (**b**). CT images revealed a mixed type of skeletal metastasis with partly osteolytic lesion predominance

**Fig. 4:**
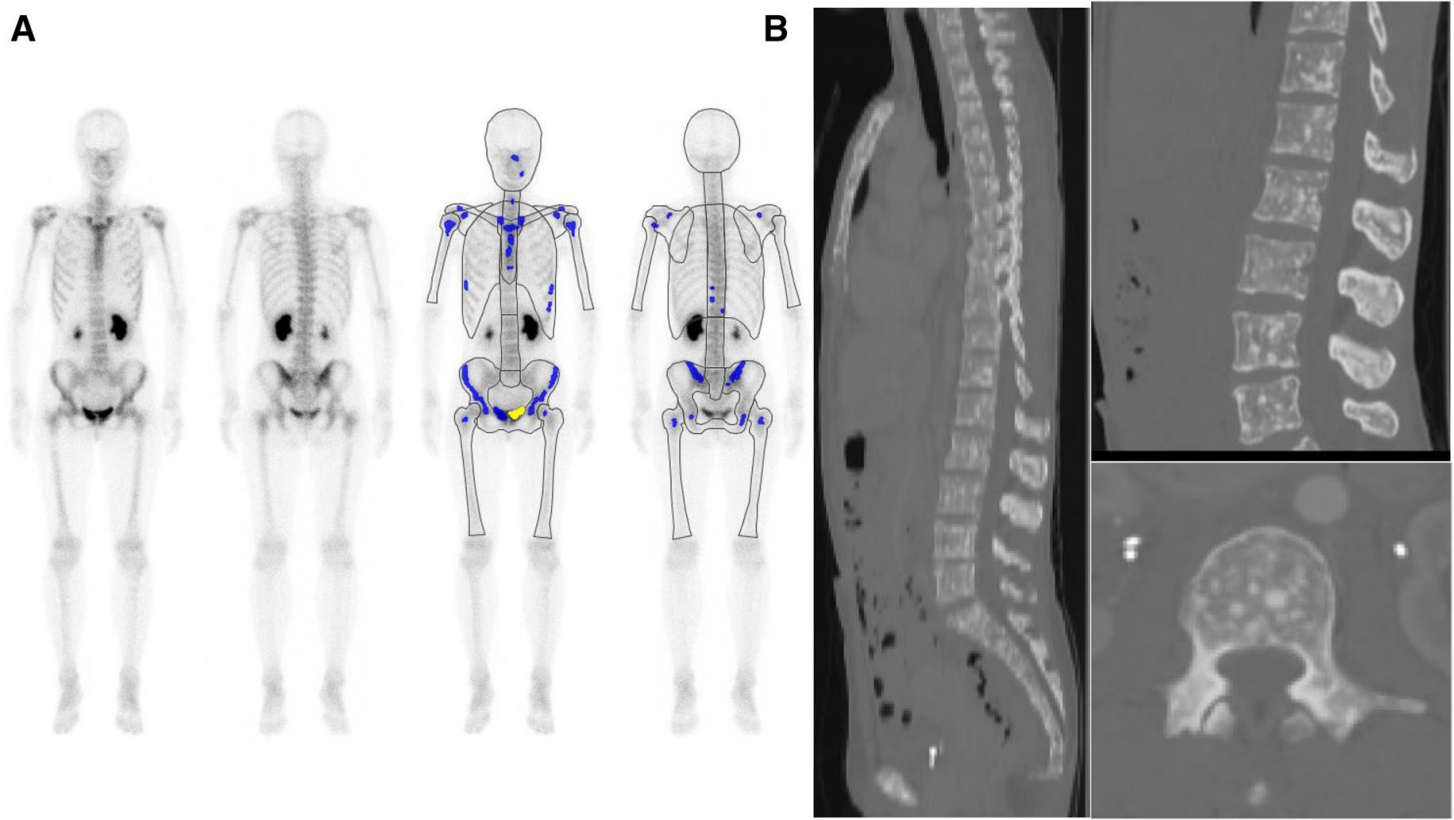
This 44-year-old female was recently diagnosed with diffuse infiltrating type-4 advanced gastric cancer (poorly differentiated signet ring cell). Her ALP and CEA values were within the normal limits (AL* p* = 207 U/l and CEA = 4.1 ng/ml). Bone scintigraphy (BS) was performed as a staging work-up study. Anterior and posterior views of whole-body BS (left) and BONENAVI processed images with artificial neural network (right) **a** show an almost normal axial skeleton uptake, no inhomogeneity of RN uptake, increased proximal extremity uptake (or contrast of proximal femur and distal portion), and normal renal uptake. The interpretation of BS was “an almost normal BS study; however, proximal extremity increased uptake was not-ruled-out to diagnose disseminated skeletal metastasis” (BS not-rule-out type). BONENAVI analysis showed ANN = 0.0 and BSI = 0.0. CT images of sagittal (left, right upper), and axial (right lower) slices (**b**). Spotty osteoblastic lesions were disseminated in the whole skeleton. Her skeletal lesions were shown to have progressed gradually in later studies

Figures 3 and 4 show patients with disseminated skeletal metastasis who showed negative BONENAVI results.

The sensitivities of the ANN value (ANN ≥ 0.5) are shown in Table 2. Total ANN sensitivity was 76% (41/54).

**Table 2:** Sensitivities of BONENAVI ANN in patients with disseminated skeletal metastasis

	Number of patients	Sensitivity*	*p* value
Primary cancer			0.023
Prostate cancer	12	100% (12/12)	
Gastric cancer	16	75% (12/16)	
Breast cancer	16	75% (12/16)	
Miscellaneous cancer	10	50% (5/10)	
CT type			0.096
Blastic	25	72% (18/25)	
Mixed	19	89% (17/19)	
Intertrabecular	10	60% (6/10)	
BS type			0.000
Typical	28	100% (28/28)	
Suspected	16	56% (9/16)	
Not-ruled-out	10	40% (4/10)	
Total	54	76% (41/54)	

*The sensitivity was calculated using ANN value ≧ 0.5 as positive case

**p values were calculated by Fisher's exact test

Regarding the types of primary cancer, patients with prostate cancer showed 100% (12/12) sensitivity, those with gastric cancer and breast cancer 75% (12/16) each, and those with miscellaneous cancers 50% (5/10).

Regarding CT type, osteoblastic type showed 72% (18/25) sensitivity, mixed type 89% (17/19), and intertrabecular type 60% (6/10). The intertrabecular type could not be found on CT scan (CT negative), and therefore, CT sensitivity was calculated as 81% (44/54).

With BS types, definite BS type showed 100% (28/28), suspected BS type 56% (9/16) and not-ruled-out BS type 40% (4/10). Because we recruited the disseminated skeletal metastasis patients based on visual BS results (all patients were diagnosed to have some abnormal findings from definite to not-ruled-out on visual interpretation), we could not calculate the sensitivity of the visual BS method.

Relation to BONENAVI (ANN and BSI values) and clinical features (primary cancers, CT pattern, and BS pattern).

To clarify the relationships of primary cancer types, CT types, and BS types to ANN value, one-way ANOVA was performed. The results are shown in Table 3.

**Table 3:** The factors related to ANN and BSI values

	Number of patients	Mean	SD	*p* value	Inter-group's *p* values		
*ANN values*							
Primary cancer							
Prostate cancer	12	0.96	0.06	0.019	vs Gastric ca: NS	vs Breast ca: NS	vs Miscellaneous ca: NS
Gastric cancer	16	0.77	0.37		vs Breast ca: NS	vs Miscellaneous ca: NS	
Breast cancer	16	0.8	0.34		vs Miscellaneous ca: NS		
Miscellaneous cancers	10	0.59	0.46				
CT types							
Osteoblastic	25	0.76	0.37	0.233	vs Mixed: NS	vs: Intertrabecular: NS	
Mixed	19	0.89	0.27		vs: Intertrabecular: NS		
Intertrabecular	10	0.67	0.42				
BS types							
Typical	28	0.97	0.09	0.002	vs Suspected: 0.01	vs: Not-ruled-out: 0.028	
Suspected	16	0.62	0.41		vs: Not-ruled-out: NS		
Not-ruled-out	10	0.54	0.44				
*BSI values*							
Kinds of cancer							
Prostate cancer	12	8.49	3.16	0.000	vs Gastric ca: NS	vs Breast ca: 0.007	vs Miscellaneous ca: 0.02
Gastric cancer	16	6.41	5.03		vs Breast ca: NS	vs Miscellaneous ca: 0.04	
Breast cancer	16	3.55	3.12		vs Miscellaneous ca: NS		
Miscellaneous cancers	10	2.29	2.97				
CT types							
Osteoblastic	25	6.09	4.72	0.247			
Mixed	19	5.16	3.76		vs Mixed: NS	vs: Intertrabecular: NS	
Intertrabecular	10	3.37	4.13		vs: Intertrabecular: NS		
BS types							
Typical	28	8.5	3.18	0.000	vs Suspected: 0.000	vs: Not-ruled-out: 0.000	
Suspected	16	1.71	2.07		vs: Not-ruled-out: NS		
Not-ruled-out	10	1.87	2.29				

*ANN* artificial neural network, *BSI* bone scan index, *SD* standard deviation, *NS* not significant

The whole analysis between ANN values and primary cancer types showed a statistically significant difference (*p* = 0.019); however, there were no significant differences among ANN values for each type of cancer.

There was no statistical difference in ANN values among CT types.

Statistically significant difference was shown in BS types (*p* = 0.002), and there was a significant difference between definite type and suspected type (*p* = 0.01), and between definite type and not-ruled-out type (*p* = 0.028).

About BSI values, kinds of cancer (*p* = 0.000) and BS patterns (*p* = 0.000) were statistically significant; however, CT types (*p* = 0.247) were not significant. Inter-group sub-analysis showed that there was a statistically significant difference between the BSI of prostate cancer and gastric cancer patients, and breast cancer and miscellaneous cancers patients. BSI values of prostate cancer and gastric cancer patients were not significantly different, and neither was there a significant difference in the BSI values between breast cancer and miscellaneous cancers.

There was a statistically significant difference between BSI values of definite BS type patients and those of others (suspected and not-ruled-out).

### Relationship between ANN value and BS findings

To investigate how BS findings are related to ANN value, one-way ANOVA was performed with ANN value as a dependent factor and BS findings as independent factors. The results are summarized in Table 4. Diffuse increased skeleton uptake (*p* = 0.001) and absent kidney uptake (*p* = 0.000) were significant factors. However, inhomogeneity of uptake (*p* = 0.45) and proximal extremity relatively increased uptake (*p* = 0.135) were not significant factors.

**Table 4:** The factors related to ANN and BSI values among BS findings

BS findings	Number of patients	Mean	SD	*p* value	Inter-group's *p* values		
ANN values							
Diffuse increased skeleton uptake							
Yes	28	0.97	0.09	0.001	vs weakly pos: 0.013		vs Normal: 0.015
Weakly yes	18	0.63	0.43		vs normal: NS		
Normal	8	0.48	0.36				
Inhomogeneity of uptake							
Inhomogenous	38	0.83	0.32	0.45	vs partially inhomogenous: NS		vs Normal: NS
Partially inhomogenous	6	0.74	0.28		vs normal: NS		
Normal	10	0.64	0.47				
Proximal extremity increased uptake							
Yes	41	0.83	0.32	0.135			
No	13	0.63	0.41				
Absent kidney							
Absent kidney	14	0.98	0.02	0.000	vs weak imaging: NS		vs normal: 0.001
Weak imaging	22	0.82	0.36		vs normal: NS		
Normal	18	0.59	0.39				
BSI values							
Diffuse increased skeleton uptake							
Yes	28	8.5	3.18	0.000	vs weakly pos: 0.000		vs normal: 0.000
Weakly yes	18	2.36	2.31		vs normal: NS		
Normal	8	0.46	0.37				
Inhomogeneity of uptake							
Inhomogenous	38	6.01	4.3	0.009	vs partially inhomogenous: NS		vs normal: NS
partially inhomogenous	6	2.22	1.88		vs normal: NS		
Normal	10	4.26	4.8				
Proximal extremity increased uptake							
Yes	41	5.96	4.23	0.036			
No	13	3.05	4.04				
Absent kidney							
Absent kidney	14	9.55	2.72	0.000	vs weak imaging: 0.001		vs normal: 0.000
Weak imaging	22	4.85	372		vs normal: NS		
Normal	18	2.43	3.46				

*ANN* artificial neural network, *BSI* bone scan index, *SD* standard deviation, *NS* not significant

We further analyzed the relation of ANN and BS findings using a multivariate logistic regression analysis. We initially used ANN ≥ 0.5; however, no patient with diffuse skeleton uptake was below 0.5 (ANN < 0.5), and the analysis was not properly performed. Therefore, we selected ANN ≥ 0.9 as the dependent factor.

This multivariate analysis showed diffuse increased skeleton uptake to be the only significant factor (*p* = 0.001), as shown in Table 5.

**Table 5:** Logistic regression analysis using ANN 0.9≧ as dependent variable

BS findings	Number of patients	Multivariate analysis		
		p value	Odds	Odds (95% CI)
Diffuse increased skeleton uptake		0.001		
Yes	28	0.000	189	10.5–3413.6
Weakly yes	18	0.064	8.75	0.88–86.6
No (base)	8			
Inhomogenous uptake		NS		
Inhomogenous	38			
Partially inhomogenous	6			
No (base)	10			
Extremity proximal increased uptake		NS		
Yes	41			
No (base)	13			
Renal uptake		NS		
Absent kidney	14			
Weak renal uptake	22			
Normal (base)	18			

*ANN* artificial neural network, *BS* bone scintigraphy, *CI* confidence interval, *NS* not significant

Regarding BSI values (one-way ANOVA), all BS factors showed statistically significant differences, but diffuse increased skeleton uptake and absent kidney sign showed strong correlations with the BSI values. On sub-group analysis, BSI values of diffuse increased skeleton uptake patients showed a significant difference compared with those of others (weak diffuse increased skeleton uptake and normal patients). BSI values of patients showing absent kidney sign were statistically significantly high compared with those of others (patients with weak renal imaging and normal renal imaging).

### ANN negative cases

Table 6 shows the list of patients who showed negative (ANN < 0.5). The types of primary cancer were variable but no prostate cancer patients were included in this list. CT types of ANN negative cases were variable: 7 were osteoblastic, 2 were mixed, and 4 were intertrabecular. No patients with definite BS type were included in the ANN negative cases. Regarding the BS findings, patients with diffuse increased skeleton uptake and absent kidney were not included in the ANN negative patient list. Many of the ANN negative patients (10/13) had received therapy for malignancy (chemotherapy and/or hormone therapy). The effect of therapy might have influenced the BONENAVI results. Indeed, 10 in 27 patients with therapy had shown ANN negative, while 3 in 27 patients without therapy showed a negative ANN result (*p* = 0.027, Fisher exact test). Patients with active cancer therapy (chemotherapy and hormone therapy) statistically significantly frequently showed negative ANN results in the BONENAVI analysis.

**Table 6:** Negative BONENAVI ANN patients with disseminated skeletal metastasis

No	Gender	Age	Primary cancer	Chemotherapy or hormone therapy	ANN	BSI	CT type	BS type	BS findings			
									Diffuse increased axial skeleton uptake	Inhomogeneity of uptake	Proximal extremity increased uptake	Absent kidney sign
1	F	34	Breast cancer	Yes	0	0	Mixed	Suspected	Weak positive	Inhomogeneity	Yes	Kidney weak
2	M	46	Maxillary cancer	No	0	0	Intertrabecullar	Suspected	No	Inhomogeneity	Yes	–
3	M	65	Pancreas cancer	Yes	0	0	Intertrabecullar	Suspected	Weak positive	Inhomogeneity	No	–
4	F	44	Gastric cancer	Yes	0	0	Blastic	Not-ruled-out	No	No	Yes	–
5	M	66	Gastric cancer	Yes	0	0	Blastic	Not-ruled-out	Weak positive	No	Yes	Kidney weak
6	M	77	Pancreas NET	Yes	0	0	Blastic	Not-ruled-out	Weak positive	No	No	Kidney weak
7	F	51	Breast cancer	Yes	0.14	0.066	Blastic	Suspected	Weak positive	Inhomogeneity	No	–
8	F	67	Gastric cancer	No	0.37	0.57	Blastic	Suspected	No	Inhomogeneity	No	–
9	F	57	Breast cancer	Yes	0.43	0.831	Blastic	Suspected	Weak positive	Inhomogeneity	No	Kidney weak
10	M	61	Lung cancer	No	0.43	0.579	Intertrabecullar	Not-ruled-out	Weak positive	Partially	Yes	–
11	F	39	Breast cancer	Yes	0.44	0.592	Mixed	Suspected	No	Inhomogeneity	Yes	–
12	M	58	Esophagus cancer	Yes	0.46	0.33	Intertrabecullar	Not-ruled-out	Weak positive	No	No	–
13	M	65	Gastric cancer	Yes	0.49	0.5	Blastic	Not-ruled-out	No	Partially	Yes	–

*BS* bone scintigraphy, *ANN* artificial neural network, *BSI* bone scan index, *NET* neuroendocrine tumor

## Discussion

The diagnosis of disseminated skeletal metastasis by BS is sometimes easily misinterpreted as normal unless the absence of renal uptake, the presence of diminished activity in the bones of the appendicular skeleton, and a high ratio of bone to soft tissue activity are recognized [1, 2]. Figures 3and 4 show patients with such a misinterpretation.

We aimed to evaluate the clinical application of the BONENAVI system on BS to resolve this problem. As shown in Table 2, patients with definite BS based on visual interpretation were correctly diagnosed (100%, 28/28) by ANN value. Patients with suspected (56%, 9/16) and not-ruled-out BS (40%, 4/10) were partially diagnosed as positive by ANN value. About half (13/16) of suspected and not-ruled-out BS patients could be identified.

Regarding CT interpretation, intertrabecular CT type means undetectable on CT study. Therefore, the sensitivity of CT for disseminated skeletal metastasis was 81% (44/54). Furthermore, in intertrabecular type (CT negative) patients, BONENAVI detected skeletal metastasis in 60% (6/10). These figures are promising that in some patients with BS difficult (not-ruled-out BS type; 4/10) and CT negative (intertrabecular type; 6/10) showed positive ANN values but were not satisfactory. Total sensitivity of ANN in disseminated skeletal metastasis (76%) was lower than the reported sensitivity for all cancers. Nakajima et al. reported that the sensitivity of ANN for all cancers was 91% [8].

According to our classification, our clinical interpretation was that patients with suspected BS could be diagnosed as having bone metastasis, but whether or not disseminated spread was present was not certain. A false-negative example of such a patient is shown in Fig. 3. Our BS clinical interpretation was that inhomogeneity of uptake and increased proximal extremity bone uptake were present, and the interpretation of this patient’s BS findings was that definite skeletal metastasis and disseminated spread were highly suspected. However, the BONENAVI result was negative (ANN = 0, BSI = 0). We speculated that the reason for the negative BONENAVI result was the nature of the skeletal metastasis, in that, even though there was a mixed CT pattern, the osteolytic component was predominant in this patient (Fig. 3). Therefore, skeletal RN uptake was not so intense, resulting in a negative ANN value.

The ANN value correlated with the BS types rather than the primary cancer or CT types (Tables 2, 3). Among the BS findings, diffuse increased skeleton uptake was best correlated to ANN rather than inhomogeneity of uptake, proximal extremity bone uptake, or absent kidney (Tables 4, 5). The diffuse increased skeleton uptake was considered the most important or impactful factor in the BONENAVI analysis.

To improve the sensitivity of BONENAVI, factors other than diffuse increased skeleton uptake, such as inhomogeneity of uptake, might be considered or included to reach the artificial intelligence of BONENAVI.

Chemotherapy and radiotherapy may influence the bone seeking RN uptake to bone. Radiation usually causes deceased bone seeking RN to bone with well demarcated margins limited to the boundaries of radiation areas [12]. The interpretation is very difficult after chemotherapy: decreased RN uptake usually implies tumor healing but sometimes a rapidly enlarging lytic lesion shows such decreased RN uptake. Conversely, an increased RN uptake usually indicates tumor progression, but in the early phase the increase may indicate a healing process mediated by the flare phenomenon [1]. The interpretation of the chemotherapeutic effect is not easy to understand. Only five of the patients in this study had irradiation therapy to bone (5/54). We abandoned the analysis of irradiation in the present study because of this low number of irradiated patients. The number of patients who underwent chemotherapy or hormone therapy (for breast cancer or prostate cancer) was not small: 13 of 16 patients with breast cancer and 4 of 12 patients with prostate cancer had received chemotherapy and/or hormone therapy prior to the BS study, while 4 of 16 patients with gastric cancer, and 6 of 10 patients with miscellaneous cancer underwent chemotherapy prior to it. Many types of cancer were included in the study. The regimen, duration, and therapeutic effects are variable, and these factors interfere with the interpretation of the results. Therefore, even though patients with systemic cancer therapy (chemotherapy and/or hormone therapy) presented a statistically significantly higher frequency of showing negative ANN results than those without systemic cancer therapy, we decided to not perform further evaluation regarding the chemotherapeutic or hormone therapy effect in this study.

A serious limitation of this retrospective study was that we started this study from the BS log. We aimed to investigate the performance of BONENAVI in disseminated skeletal metastasis, and we regarded a BS study to be essential in this study. However, there is a possibility that BS was not performed (or not ordered) in all patients with disseminated skeletal metastasis. We recorded the BS log when any suspicious or not-ruled-out findings in BS study were identified. However, there is a possibility that patients with normal BS finding might have had disseminated skeletal metastasis. Therefore, we could not calculate the sensitivity of visual BS interpretation. We established a diagnosis of disseminated skeletal metastasis when at least two imaging modalities were consistent with the diagnosis. However, the sensitivities and specificities of each modality are not 100%, that is, currently there is no gold standard to diagnose disseminated skeletal metastasis.

Another drawback was that the diagnosis of disseminated skeletal metastasis was performed clinically; that is, no histopathological confirmation was carried out.

## Conclusion

The BONENAVI CAD system was partially helpful in diagnosing disseminated skeletal metastasis, but the sensitivity of BONENAVI was not sufficient (76%) and underestimated disseminated skeletal metastasis. BONENAVI results may underestimate the disseminated skeletal metastasis by the present version.
